# Effect of remote ischemic conditioning on the immune-inflammatory profile in patients with traumatic hemorrhagic shock in a randomized controlled trial

**DOI:** 10.1038/s41598-023-33681-3

**Published:** 2023-04-29

**Authors:** C. H. Leung, S. B. Rizoli, S. Trypcic, S. G. Rhind, A. P. Battista, M. Ailenberg, O. D. Rotstein

**Affiliations:** 1 The Keenan Research Centre for Biomedical Science and the Department of Surgery, St. Michael’s Hospital, Unity Health Toronto, 30 Bond Street, Li Ka Shing Knowledge Institute 3-305, Toronto, ON M5B 1W8 Canada; 2 The Defence Research and Development Canada, Toronto Research Centre, Toronto, Canada; 3 Department of Surgery, University of Toronto, Toronto, Canada

**Keywords:** Disease prevention, Interleukins, Chemokines

## Abstract

Resuscitation induced ischemia/reperfusion predisposes trauma patients to systemic inflammation and organ dysfunction. We investigated the effect of remote ischemic conditioning (RIC), a treatment shown to prevent ischemia/reperfusion injury in experimental models of hemorrhagic shock/resuscitation, on the systemic immune-inflammatory profile in trauma patients in a randomized trial. We conducted a prospective, single-centre, double-blind, randomized, controlled trial involving trauma patients sustaining blunt or penetrating trauma in hemorrhagic shock admitted to a Level 1 trauma centre. Patients were randomized to receive RIC (four cycles of 5-min pressure cuff inflation at 250 mmHg and deflation on the thigh) or a Sham intervention. The primary outcomes were neutrophil oxidative burst activity, cellular adhesion molecule expression, and plasma levels of myeloperoxidase, cytokines and chemokines in peripheral blood samples, drawn at admission (pre-intervention), 1 h, 3 h, and 24 h post﻿-admission. Secondary outcomes included ventilator, ICU and hospital free days, incidence of nosocomial infections, 24 h and 28 day mortality. 50 eligible patients were randomized; of which 21 in the Sham group and 18 in the RIC group were included in the full analysis. No treatment effect was observed between Sham and RIC groups for neutrophil oxidative burst activity, adhesion molecule expression, and plasma levels of myeloperoxidase and cytokines. RIC prevented significant increases in Th2 chemokines﻿ TARC/CCL17 (P < 0.01) and MDC/CCL22 (P < 0.05) at 24 h post-intervention in comparison to the Sham group. Secondary clinical outcomes were not different between groups. No adverse events in relation to the RIC intervention were observed. Administration of RIC was safe and did not adversely affect clinical outcomes. While trauma itself modified several immunoregulatory markers, RIC failed to alter expression of the majority of markers. However, RIC may influence Th2 chemokine expression in the post resuscitation period. Further investigation into the immunomodulatory effects of RIC in traumatic injuries and their impact on clinical outcomes is warranted.

ClinicalTrials.gov number: NCT02071290.

## Introduction

Traumatic injuries continue to be a leading cause of mortality worldwide^1^ despite significant advances in the comprehensive treatment of the trauma patient. Organ dysfunction observed in severely injured resuscitated patients, is, in part, attributed to the development of systemic immune dysfunction^2,3^. The pathophysiological process of ischemia/reperfusion occurring in patients resuscitated from hemorrhagic shock initiates a cascade of cellular events leading to an altered immune status, and includes generation of oxidative stress, release of inflammatory cytokines, and the priming of immune cells^2,4,5^. The attendant phenotypic switch from Th1 to Th2 adaptive immunity post resuscitation is associated with immunosuppression^6^. Together these events predispose trauma patients to the systemic inflammatory response syndrome, acute respiratory distress syndrome, infections, and multiple organ dysfunction^7^.

An intervention that has been shown to lessen the magnitude of ischemia/reperfusion injury is called Remote Ischemic Conditioning (RIC)^8^. RIC is a non-invasive therapeutic procedure consisting of repetitive applications of brief ischemia/reperfusion to a distal limb by inflating and deflating a pressure cuff. In experimental studies, antecedent administration of RIC ameliorates distant organ ischemia/reperfusion injury. RIC has also been shown to exert anti-inflammatory effects when applied in healthy volunteers^9,10^. These findings have been translated to clinical trials to show a beneficial effect of RIC in renal^11^ and cardiac^12^ protection. There has been considerable study of the mechanisms underlying the salutary effects of RIC on distant organ injury. Both humoral and neuronal pathways have been invoked, often with both being involved and frequently interacting, in mediating the transfer of the upstream signal i.e. RIC to downstream organ protection^13,14^. Further, both may be involved in the downstream effect leading to organ protection. It has also been reported that the elaboration of reactive oxygen species by the preconditioning intervention might exert protection through activation of redox-sensitive events^15^.

In the trauma setting, Joseph et al. demonstrated that RIC applied in patients following traumatic brain injury exerted neuroprotective effects, as determined by a reduction of neuronal biomarkers of acute brain injury in the blood^16^. In animal models of hemorrhagic shock/resuscitation, we and others have shown the protective effect of RIC in preventing organ injury in the liver^17^, lung^17,18^, brain^19^ and heart^20^. However, its ability to more broadly modulate immune function in trauma populations sustaining hemorrhagic shock, has not been evaluated. Whether RIC applied post-injury in trauma patients can initiate its upstream signaling and downstream protective effect, in which hemorrhage has led to hemodynamic instability, inflammatory responses, immune cell alterations, platelet dysfunction, and coagulopathy, remains to be determined.

We hypothesized that RIC may suppress the inflammatory response associated with traumatic resuscitated hemorrhagic shock. The major objective of this clinical trial is to investigate the potential immunomodulatory effects of posttraumatic application of RIC in trauma patients sustaining hemorrhagic shock by evaluating markers of neutrophil activation and systemic inflammation during the acute phase of injury.

## Methods

### Patient population and study design

A prospective, single-centre, randomized, controlled double-blind clinical trial was conducted at St. Michael’s Hospital, Unity Health Toronto, a Level 1 provincial trauma centre, to investigate the immunomodulatory effects of Remote Ischemic Conditioning in trauma patients (age > 16) sustaining blunt or penetrating injuries and in hemorrhagic shock. The trial is registered on ClinicalTrials.gov (Identifier: NCT02071290) on 25/02/2014. Hemorrhagic shock was defined as one or more recorded episode of systolic blood pressure under 90 mmHg at any time prior to enrolment with an identified source of blood-loss. The RIC procedure was to be completed within 4 h of injury to be eligible. Major exclusions included patients who were on anticoagulant medication, pregnancy, recorded systolic blood pressure of above 200 mmHg, burns, absence of vitals, or ongoing cardiopulmonary resuscitation. Patients were enrolled by research coordinator through deferred consent as permitted by The Tri-Council Policy Statement on the Ethical Conduct of Research with Humans (TCPS2) and informed consent was obtained from all participants by the substitute decision maker within 24 h of enrolment. After randomization, blood samples were collected into citrate, EDTA, and heparin vacutainers at Admission (prior to initiation of Sham or RIC intervention), and at 1, 3, and 24 h after admission. These timepoints were chosen to reflect the biphasic cytoprotection afforded by RIC^21^; i.e. the early cytoprotection that lasts up to ~ 3 h after RIC (first window of protection) and the late cytoprotection that emerges ~ 24 h after RIC (second window of protection). Fresh heparinized whole blood samples for flow cytometric analysis were stained within 1 h of collection. Admission data including patient demographics, diagnostic laboratory values, and outcomes were collected.

As healthy controls, an additional 10 volunteers were enrolled and subjected to identical blinding, exclusion criteria, and randomization procedure as trauma patients to receive either Sham (n = 5) or RIC intervention (n = 5). The combined Admission values from the 10 volunteers served as Healthy Control comparisons to trauma patients.

The study was approved by the Research Ethics Board at St. Michael’s Hospital, Unity Health Toronto (14-047). All methods and procedures were performed in accordance with guidelines and regulations of The Tri-Council Policy Statement on the Ethical Conduct of Research with Humans (TCPS2).

### Randomization and blinding

Patients were randomized in a 1:1 ratio to receive either a sham intervention or RIC intervention by a sealed envelope containing the treatment allocation. The randomization schedule was generated in R using random permuted blocks of sizes 2 and 4 by a statistician who was not involved in the trial. The study investigators and research staff who performed laboratory tests were blinded to the treatment allocation.

### Interventions

RIC was performed using a pneumatic tourniquet (Zimmer A.T.S. 3000, Zimmer, Mississauga, ON, Canada) by inflation of a sterile pressure cuff (Vari Fit Contour Tourniquet Cuff, Delfi Medical Innovations, Vancouver, BC, Canada) on the thigh at 250 mmHg for 5 min followed by deflation at 0 mmHg for 5 min and repeated for four cycles. The Sham intervention consisted of application of the thigh cuff with the inflation period set to 0 mmHg. Patients were observed for any local events related to inflation of the cuff.

### Outcomes

The primary outcomes were neutrophil oxidative burst activity, neutrophil cell surface adhesion molecule expression and plasma levels of cytokines and chemokines in blood samples taken at admission (pre-intervention), and one, three, and 24 h post admission. Secondary clinical outcomes assessed at 28 days included ventilator free days, ICU free days, hospital free days, nosocomial infections, and 24 h and 28 day mortality.

### Feasibility outcomes

Outcomes for the feasibility of RIC include the ability to enrol eligible patients in the trauma bay, application and completion of RIC, interruption of RIC cycles, total duration of RIC, and duration from time of injury to application of RIC.

### Neutrophil oxidative burst activity

The intracellular oxidative burst capacity of neutrophils was measured using commercially available flow cytometric kit (PHAGOBURST; Glycotope Biotechnology, Heidelberg, Germany). Heparinized whole blood (100 µl) was incubated with either wash buffer (unstimulated control), stimulated with PMA (1.35 µM) or fMLP (0.83 µM) for 10 min at 37 °C followed by incubation with DHR-123 substrate for 10 min at 37 °C. Erythrocytes were lysed with the addition of Lysing Solution (Glycotope Biotechnology). Cells were centrifuged at 250×*g* for 5 min, washed once, and stained with CD14-Alexa Fluor 647 (clone MφP9; BD Biosciences) and CD16-eFluor 450 (clone CB16; Affymetrix) antibodies at 4 °C for 30 min. Stained cell suspensions were acquired using LSRFORTESSA X-20 flow cytometer (BD Biosciences) within 30 min of staining on 10,000 CD16^+^CD14^−^ neutrophils. Unstained controls in separate tubes were used to control for autofluorescence. Data were analyzed by FACSDIVA (BD Biosciences) and the Median Fluorescence Intensity (MFI) for Rho-123 was recorded.

### Neutrophil cell-surface adhesion molecule

Neutrophil cell surface adhesion molecules were assessed with antibodies staining for CD11b-PE (clone CBRM1/5; Affymetrix) and CD62L-FITC (clone DREG-56; BD Biosciences). Antibodies staining for CD14-Alexa Fluor 647 (clone MφP9; BD Biosciences) and CD16-eFluor 450 (clone CB16; Affymetrix) were used to facilitate separation of monocyte and neutrophil populations respectively. Heparinized whole blood (100 µl) was incubated with either wash buffer (unstimulated control), PMA (1.35 µM), or fMLP (0.83 µM) for 10 min at 37 °C followed by staining with antibodies for 20 min at room temperature in the dark. Erythrocytes were lysed with the addition FACS Lysing Solution (BD Biosciences). Cells were spun at 250×*g* for 5 min, washed once, and resuspended in 1% BSA-PBS. Stained cell suspensions were acquired using LSRFORTESSA X-20 flow cytometer (BD Biosciences) on 10,000 CD16^+^CD14^−^ neutrophils. Unstained controls in separate tubes were used to control for autofluorescence. Data were analyzed by FACSDIVA (BD Biosciences) and the Median Fluorescence Intensity (MFI) for CD11b and CD62L were measured.

### Plasma cytokine, chemokine, and myeloperoxidase

Whole blood collected in EDTA vacutainers were centrifuged at 2500*g* for 15 min to acquire plasma and aliquots were frozen in − 80 °C until analysis. Plasma levels of a panel of 30 cytokines and chemokines were measured using an electrochemiluminescence-based multiplex sandwich immunoassay (V-PLEX Human Cytokine 30-Plex Kit, Meso Scale Diagnostics). Myeloperoxidase (MPO) was measured using Human Myeloperoxidase Kit (Meso Scale Diagnostics). Analytes were analyzed on a Sector Imager 6000 (Meso Scale Diagnostics) according to manufacturer’s instructions. The cytokine and chemokines assayed from this kit include Eotaxin, Eotaxin-3, granulocyte–macrophage colony stimulating factor (GM-CSF), Interferon γ (IFN-γ), interleukin (IL)-10, IL-12/IL-23p40, IL-12p70, IL-13, IL-15, IL-16, IL-17A, IL-1α, IL-1β, IL-2, IL-4, IL-5, IL-6, IL-7, IL-8, IL-8 (HA), Inducible protein-10 (IP-10), monocyte chemoattractant protein-1 (MCP-1), MCP-4, Macrophage-derived chemokine (MDC/CCL22), Macrophage inflammatory protein-1α (MIP-1α), MIP-1β, Thymus and Activation Regulated chemokine (TARC/CCL17), tumour necrosis factor α (TNF-α), TNF-β, Vascular endothelial growth factor a (VEGF-a). Samples were measured in duplicate and results were used for statistical analysis only if plasma levels fell within the lower and upper limits of detection, were detectable in ≥ 80% of analyzed samples, and had a percentage coefficient of variance < 25% between replicates. As a result, IL-1α, IL-1β, IL-2, IL-4, IL-8 (HA), IL-12p70, IL-13, MIP-1α, GM-CSF, Eotaxin-3, and TNF-β were excluded from analysis.

### Statistical analysis

Sample size calculation was based on the variance from previous clinical trial in a similar cohort of trauma patients (SD of 27.2 for CD11b and SD of 20.1 for CD62L). A sample size of 20 for each group gives 80% power to detect a difference of 24.7 for CD11b or 18.2 for CD62L. Such differences represent clinically important differences in neutrophil activation. Data analysis was performed using SPSS version 23 (SPSS, Chicago, Illinois). Patient admission characteristics and outcomes were compared by Mann–Whitney *U* or χ^2^ test where appropriate. A linear mixed model was used for repeated measures analysis. Post hoc comparisons were performed on the Estimated Marginal Means adjusting for the trauma patients’ admission value, Injury Severity Score, base deficit, age, and time to intervention from injury. Post hoc comparisons between healthy controls were adjusted for Admission values. Multiple comparisons were subjected to Bonferroni correction. Comparisons between trauma patients and healthy controls were performed by one-way analysis of variance followed by Dunnett’s post hoc test. Data for linear mixed model and analysis of variance were tested for normality using Shapiro–Wilk test and non-parametric data were log transformed prior to analysis. Continuous data are expressed as median (interquartile range). A P value of < 0.05 was considered statistically significant.

## Results

**Figure 1:**
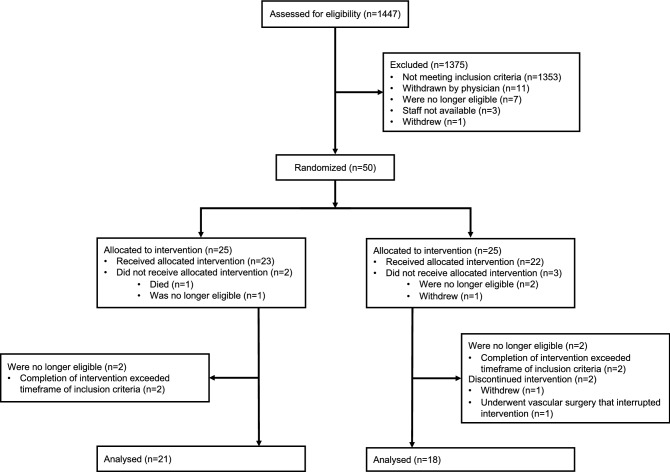
Patient screening and enrollment statistics.

A total of 1447 trauma patients admitted to St. Michael’s Hospital were screened for eligibility between May 2015 to October 2016, of whom 72 patients met enrolment criteria (Fig. 1). Of the 72 eligible patients, 50 patients were enrolled and randomized between Sham (n = 25) and RIC interventions (n = 25). 11 of the 50 enrolled patients became ineligible after randomization. A total of 39 patients were included in the final analysis: 21 in the Sham group and 18 in the RIC group. Patient pre-hospital and admission clinical characteristics were not significantly different between Sham and RIC groups (Table 1).

**Table 1:** Patient prehospital and admission demographics and clinical characteristics.

	Sham	RIC	P value
Age	37 ± 17	40 ± 17	0.67
Gender	76% male	78% male	0.91
No. (%) of penetrating, blunt trauma	14 (67%), 7 (33%)	9 (50%), 9 (50%)	0.29
Injury Severity Score	16 (6, 26)	17 (10, 25)	0.43
Apache II	10 (6, 19)	10 (6, 16)	0.70
SOFA	8 (5, 9)	6 (4, 8)	0.45
Scene systolic pressure	88 (72, 118)	80 (68, 94)	0.38
Scene diastolic pressure	62 (42, 85)	60 (49, 64)	0.38
Scene Glasgow Coma Scale	15 (14, 15)	15 (14, 15)	0.58
Scene pulse	110 (78, 130)	99 (75, 115)	0.33
Scene respiratory rate	20 (16, 24)	20 (13, 23)	0.75
Admission systolic pressure	106 (83, 126)	107 (88, 118)	0.99
Admission diastolic pressure	59 (50, 75)	62 (56, 77)	0.43
Admission Glasgow Coma Scale	15 (14, 15)	15 (7, 15)	0.71
Admission pulse	110 (80, 139)	87 (79, 110)	0.07
Admission respiratory rate	20 (17, 23)	20 (16, 23)	0.67
Base	− 4.8 (− 7.2, − 2.5)	− 3.7 (− 6.3, − 2.0)	0.39
Lactate	6 (2.8, 7.7)	4.1 (2.4, 6.3)	0.28
PTT	27 (26, 30)	27 (25, 31)	0.48
INR	1.10 (1.05–1.23)	1.14 (1.09–1.34)	0.31
RBC, median (IQR)	4 (3, 10)	4 (2, 10)	0.72
Platelets, median (IQR)	0 (0, 1)	0 (0, 1)	0.35
Fresh frozen plasma, median (IQR)	2 (0, 7)	1 (0, 5)	0.4
Cryoprecipitate, median (IQR)	0 (0, 2)	0 (0, 2)	0.83
Time to intervention from injury (Min)	97 (70, 139)	126 (82, 143)	0.21

Values represent median (interquartile range) or number (percentile).

Clinical outcomes including ventilator free days, ICU free days, hospital free days, nosocomial infections, and mortality were not significantly different between Sham and RIC groups (Table 2). No adverse events were observed related to the administration of the RIC intervention.

**Table 2:** Patient clinical outcomes.

	Sham	RIC	P value
Ventilator free days	27 (24, 28)	26 (24, 28)	0.323
ICU free days	27 (23, 28)	25 (18, 27)	0.287
Hospital free days	24 (11, 26)	16 (7, 22)	0.151
Nosocomial infections	5 (24%)	6 (33%)	0.510
24 hour mortality	1 (5%)	0	0.348
28 day mortality	1 (5%)	1 (6%)	0.911

Values represent median (interquartile range) or number (percentile).

### Feasibility of RIC

Of the 25 enrolled patients in the Sham arm, the intervention was completed in 23 patients: 2 of the patients did not have the intervention initiate due to death of one patient after enrolment and another patient was not able to initiate the intervention within the inclusion period time frame. Of the 25 enrolled patients in the RIC arm, 20 patients completed the RIC intervention. The intervention was not initiated in 3 patients due to the inability to draw admission blood from one patient, one patient opted out of the study, and one patient was not able to initiate RIC within the inclusion period time frame. Two of RIC patients had the intervention started but prematurely terminated before all the cycles were completed due to the necessity to perform vascular surgery on the leg which RIC was performed and one patient had the intervention stopped during the first ischemic cycle due to discomfort. In addition, 4 patients from the Sham arm and 5 patients from the RIC arm had the RIC cycles interrupted due to transfer of the patients out to other departments including the Intensive Care Unit, CT Scan, or Operating Room where the interventions were resumed following transfer. The mean time for the completion of the intervention for patients who had the intervention interrupted was not significantly different between the Sham intervention (68 ± 22 min) and the RIC intervention (68 ± 21 min, P = 0.99).

The mean duration from injury to application of the Sham intervention (114 ± 53 min) was not different in comparison to the RIC intervention (130 ± 50 min, P = 0.31). The mean duration from the time of injury to completion of the intervention was also not significantly different between Sham (154 ± 56 min) and RIC (181 ± 46 min, P = 0.09) patients. Both arms had two patients in which completion of the intervention exceeded the 4 h inclusion period time frame. Significant positive correlations were observed between APACHE II score (Supplementary Fig. 1A) and the Injury Severity Score (Supplementary Fig. 1B) to the duration of time from injury to initiation of the intervention, suggesting the complexity of injuries and the patients’ pathological conditions contribute to the variability and delay in the application of RIC.

The median duration for transport time from the arrival to scene of injury to admission was 25 min (IQR: 15, 34) by ambulance and 70 min (IQR: 51, 85) by helicopter. Those patients who had prolonged transport time were due to transfers from other hospitals. These data suggest there is potentially sufficient time for pre-hospital application of RIC during EMS transport.

### Neutrophil oxidative burst, adhesion molecules, and MPO (Figs. 2 and 3)

**Figure 2:**
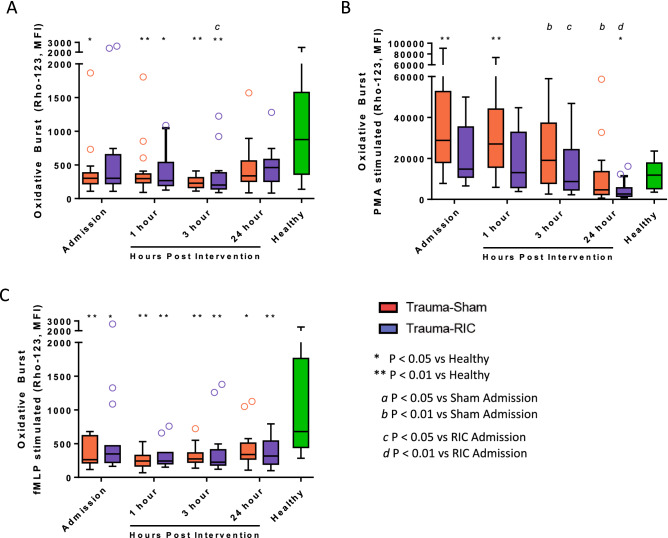
Neutrophil oxidative burst capacity from trauma patients expressed in median fluorescence intensity (MFI). (A) Unstimulated cells. (B) PMA stimulated cells. (C) fMLP stimulated cells. Lines represent the median and interquartile range. Red open circle = Trauma-Sham. Blue open circle = Trauma-RIC. Green open circle = Healthy Controls. ^a^P < 0.05 vs Sham Admission. ^b^P < 0.01 vs Sham Admission. ^c^P < 0.05 vs RIC Admission. ^d^P < 0.01 vs RIC Admission. *P < 0.05 vs Healthy Controls. **P < 0.01 vs Healthy Controls.

**Figure 3:**
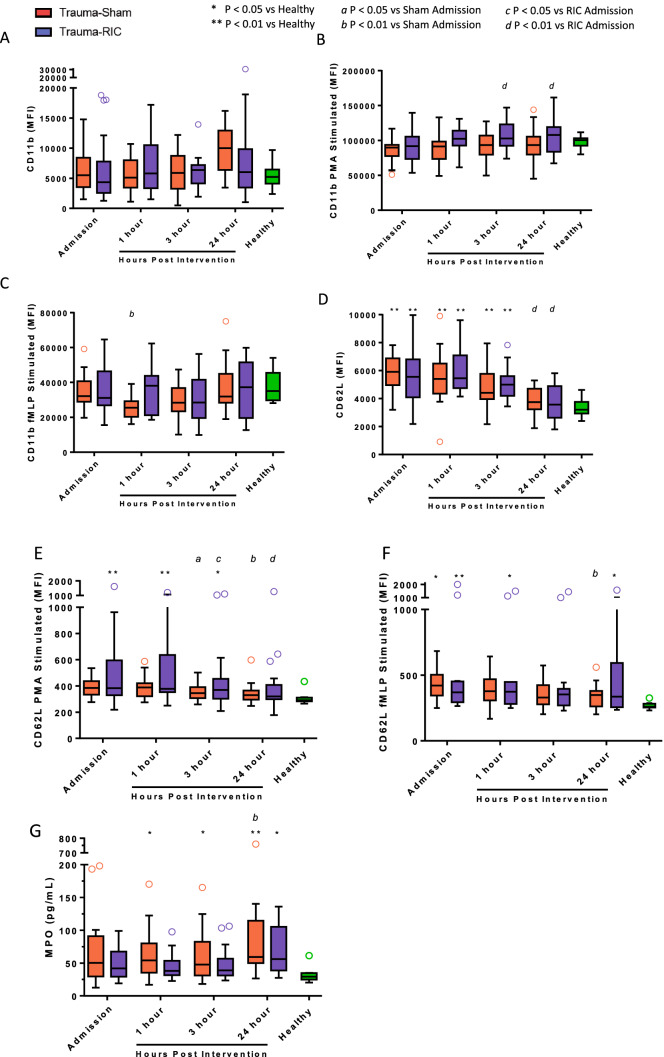
Neutrophil cell surface adhesion molecule expression from trauma patients expressed in median fluorescence intensity (MFI). (A) unstimulated CD11b expression. (B) PMA stimulated CD11b expression. (C) fMLP stimulated CD11b expression. (D) unstimulated CD62L expression. (E) PMA stimulated CD62L expression. (F) fMLP stimulated CD62L expression. (G) Plasma levels of Myeloperoxidase (MPO) in trauma patients. Red open circle = Trauma-Sham. Blue open circle = Trauma-RIC. Green open circle = Healthy Controls. Lines represent the median and interquartile range. ^a^P < 0.05 vs Sham Admission. ^b^P < 0.01 vs Sham Admission. ^c^P < 0.05 vs RIC Admission. ^d^P < 0.01 vs RIC Admission. *P < 0.05 vs Healthy Controls. **P < 0.01 vs Healthy Controls.

Unstimulated neutrophil ROS production was lower at Admission in the Sham (P < 0.05) and RIC groups (P = 0.079) and remained significantly lower at 1 and 3 h compared to healthy controls. By 24 h, ROS did not differ from healthy controls (Fig. 2A). In RIC treated patients, unstimulated ROS production decreased significantly at 3 h post intervention in comparison to Admission values. PMA-stimulated neutrophil ROS from trauma patients exposed to Sham intervention was significantly higher than healthy controls on admission and gradually fell, becoming no different from healthy controls by 3 h. ROS elaborated by PMA stimulated neutrophils from RIC-treated patients similarly fell over the first 24 h and at no time point did this significantly differ from Sham treated trauma patients. (Fig. 2B). Patients receiving the RIC intervention had significantly lower PMA stimulated ROS production at 24 h post intervention in comparison to healthy controls, a finding that was also seen in the Sham group, although not statistically significant (P = 0.408). Neutrophil ROS in response to fMLP was significantly lower at all time points in comparison to healthy controls for both Sham and RIC groups (Fig. 2C), but did not differ from each other.

The cell surface expression of neutrophil CD11b was not significantly different between Sham or RIC-treated trauma patients and healthy controls under unstimulated, PMA, or fMLP conditions (Fig. 3A–C, respectively) at any time point. PMA stimulated CD11b expression was higher at 3 and 24 h post intervention for the RIC group in comparison to Admission values (Fig. 3B), a finding not seen in Sham patients. fMLP stimulated CD11b expression was transiently decreased in the Sham group, at 1 h post intervention in comparison to Admission values (Fig. 3C).

Cell surface CD62L expression was increased in trauma neutrophils at Admission in both Sham and RIC groups, compared to healthy controls and gradually returned towards levels in healthy control by 24 h post intervention (Fig. 3D). Stimulation with PMA and fMLP reduced surface CD62L (Fig. 3E,F). No significant differences between the Sham and RIC groups were detected in unstimulated CD62L expression or after PMA or FMLP stimulation.

Sham-treated trauma patients had a significant increase in MPO (Fig. 3G) at 1 and 3 h after admission compared to healthy controls, a finding not observed at comparable time points in RIC-treated patients. By 24 h post admission, plasma MPO was elevated in both sham and RIC patients compared to healthy controls. No differences were observed between Sham and RIC-treated patients at any of the time point studied.

### Effect of RIC on plasma cytokines and chemokines

**Figure 4:**
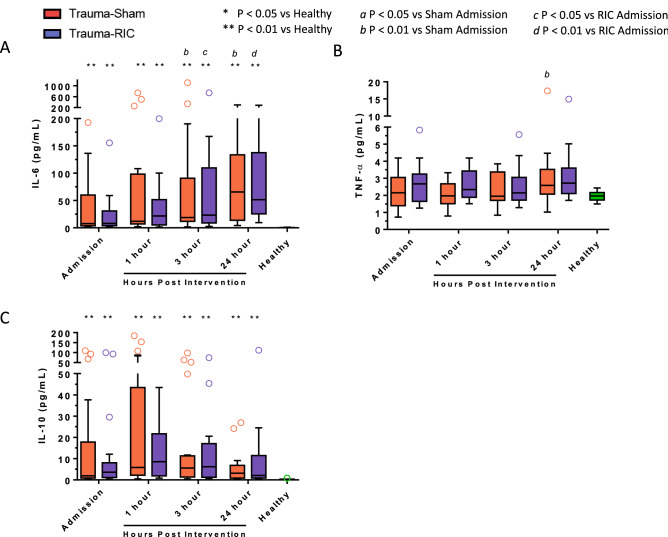
Plasma cytokine levels in trauma patients. (**A**) IL-6, (**B**) TNF-α, (**C**) IL-10. Red open circle = Trauma-Sham. Blue open circle = Trauma-RIC. Green open circle = Healthy Controls. Lines represent the median and interquartile range. ^a^P < 0.05 vs Sham Admission. ^b^P < 0.01 vs Sham Admission. ^c^P < 0.05 vs RIC Admission. ^d^P < 0.01 vs RIC Admission. *P < 0.05 vs Healthy Controls. **P < 0.01 vs Healthy Controls.

Increased plasma levels of IL-6 were observed in trauma patients compared to healthy controls upon admission and progressively increased over the 24 h study period in both Sham and RIC patients (Fig. 4A). Levels of TNF-α did not differ between trauma patients and healthy controls, either at the time of admission or over the 24 h course of study (Fig. 4B). IL-10 was elevated in both Sham and RIC upon admission in trauma patients compared to healthy controls and did not change over the 24 h post-intervention period (Fig. 4C). RIC did not significantly influence the plasma levels of IL-6, TNF-α, and IL-10 in comparison to Sham patients at any of the time points studied.

Cytokines including IL-15, IL-16, and IL-17A (Supplement Fig. 2A–C) were increased following traumatic injuries in comparison to healthy controls but did not differ between Sham and RIC treated patients. Levels of IL-12p40 (Supplement Fig. 2D) were decreased at 24 h post intervention in the Sham group in comparison to healthy controls. Levels of IFN-γ (Supplement Fig. 2E), IL-5 (Supplement Fig. 2F), IL-7 (Supplement Fig. 2G) were not different between trauma patients and healthy controls. VEGF (Supplement Fig. 2H) was significantly increased at 24 h in Sham patients in comparison to Admission values and healthy controls, but this increase was not observed in RIC patients. Application of RIC did not alter the plasma levels of molecules associated with innate or adaptive immunity compared to Sham treatment.

**Figure 5:**
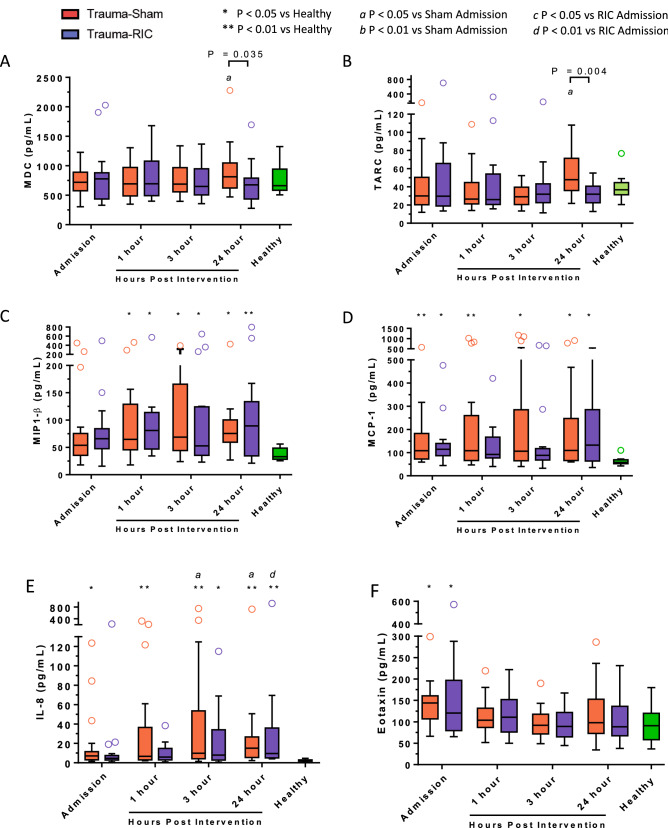
Plasma chemokine levels in trauma patients. (**A**) MDC, (**B**) TARC, (**C**) MCP-1β, (**D**) MCP-1, (**E**) IL-8, (**F**) Eotaxin. Red open circle = Trauma-Sham. Blue open circle = Trauma-RIC. Green open circle = Healthy Controls. Lines represent the median and interquartile range. ^a^P < 0.05 vs Sham Admission. ^d^P < 0.01 vs RIC Admission. *P < 0.05 vs Healthy Controls. **P < 0.01 vs Healthy Controls.

Traumatic injuries induced changes in a number of chemokines (Fig. 5). MDC/CCL22 (Fig. 5A) and TARC/CCL17 (Fig. 5B), both of which are chemokines for Th2 signaling, were significantly elevated at 24 h post intervention in Sham patients compared to admission. This rise was not observed in patients who received RIC and at the 24 h timepoint, there was a significant difference between sham and RIC treated trauma patients (MDC: P < 0.05; Cohen’s *d* = 0.54), TARC: P < 0.01; Cohen’s *d* = 0.58). Plasma levels of MIP-1β (Fig. 5C), MCP-1 (Fig. 5D), IL-8 (Fig. 5E), and Eotaxin (Fig. 5F) were significantly increased after trauma in comparison to healthy controls but there was no difference between Sham and RIC groups. Levels of IP-10 (Supplement Fig. 3A) and MCP-4 (Supplement Fig. 3B) were not significantly different between trauma patients and healthy controls.

### Effect of RIC in neutrophil oxidative burst activity and adhesion in healthy controls

As a positive control for the effectiveness of the RIC procedure used in these studies, we performed this protocol in healthy controls and measured its effect on neutrophil ROS and adhesion molecules. Application of RIC in healthy controls significantly decreased unstimulated neutrophil ROS production in comparison to Sham healthy controls at 1, 3, and 24 h post intervention (Table 3). PMA stimulated ROS production was also significantly lower in RIC treated healthy controls at 24 h post intervention (Table 3). ROS production after stimulation with fMLP was not significantly different between Sham and RIC healthy controls.

**Table 3:** Neutrophil oxidative burst and adhesion molecule expression in healthy controls.

	Admission	1 h	3 h	24 h
Oxidative burst (MFI)				
Healthy-Sham	1058 (188–2009)	1223 (346–2002)	897 (167–1970)	799 (283–3012)
Healthy-RIC	696 (441–1397)	415 (174–589)**^d^	232 (139–604)**^d^	333 (263–1248)**^d^
Oxidative burst PMA stimulated (MFI)				
Healthy-Sham	5810 (3566–14,964)	3403 (2574–10,640)	2929 (2252–8316)	10,386 (4174–16,635)
Healthy-RIC	14,201 (11,869–20,106)	9376 (4643–17,183)	7401 (6002–10,162)^c^	9768 (4347–10,706)^c^*
Oxidative burst fMLP stimulated (MFI)				
Healthy-Sham	1314 (314–2093)	1578 (542–1981)	1443 (301–2603)	1347 (367–2598)
Healthy-RIC	666 (571–1461)	507 (292–1450)	534 (282–1741)	482 (351–2023)
CD11b (MFI)				
Healthy-Sham	4621 (3409–9161)	7010 (3758–8853)	5853 (3855–11,016)	6659 (5054–12,674)^a^
Healthy-RIC	6265 (3925–6463)	6182 (3164–8488)	4383 (3528–5741)^c^*	4581 (3381–9863)^c^*
CD11b PMA (MFI) stimulated				
Healthy-Sham	100,706 (95,901–111,273)	119,293 (98,864–125,403)	111,355 (93,781–124,044)	99,262 (87,213–112,462)
Healthy-RIC	95,307 (86,399–100,673)	92,965 (78,433–106,338)	98,377 (82,617–104,146)	99,570 (92,711–104,278)
CD11b fMLP stimulated (MFI)				
Healthy-Sham	44,729 (35,827–50,166)	39,884 (37,919–50,432)	42,945 (26,624–53,510)	46,781 (27,915–51,094)
Healthy-RIC	30,806 (29,306–34,040)	29,087 (24,524–34,587)	33,103 (24,859–40,108)	29,281 (25,205–35,708)
CD62L (MFI)				
Healthy-Sham	3140 (2901–3792)	3226 (2842–3814)	2944 (2259–3398)	2732 (2336–3829)
Healthy-RIC	3268 (2696–4008)	3235 (2759–4141)	3257 (2641–3968)	3083 (2074–4126)
CD62L PMA stimulated (MFI)				
Healthy-Sham	287 (283–374)	308 (296–376)	327 (285–391)	334 (285–387)
Healthy-RIC	295 (276–307)	296 (271–304)	309 (290–321)	309 (276–336)
CD62L fMLP stimulated (MFI)				
Healthy-Sham	285 (242–300)	267 (238–307)	258 (237–314)	265 (235–301)
Healthy-RIC	269 (258–285)	260 (249–270)	255 (251–285)	269 (242–273)

Values represent median (interquartile range). ^a^P < 0.05 vs Sham Admission. ^c^P < 0.05 vs RIC Admission. ^d^P < 0.01 vs RIC Admission. *P < 0.05 vs Sham. **P < 0.01 vs Sham.

RIC significantly decreased neutrophil expression of CD11b at 3 and 24 h post intervention in comparison to Sham healthy controls (Table 3). Expression of CD11b after stimulation with PMA or fMLP was not significantly different between Sham and RIC treated healthy controls (Table 3). RIC did not significantly influence expression of CD62L in neutrophils under unstimulated, PMA, or fMLP stimulated conditions in healthy controls (Table 3).

### Plasma cytokines, chemokines, and MPO in healthy controls

Plasma levels of cytokines, chemokines, and MPO were not significantly different between healthy controls receiving the Sham and RIC intervention in each of the time points measured after the intervention (Supplementary Table 1).

## Discussion

Dysregulated immuno-inflammatory responses may contribute to adverse outcomes following traumatic hemorrhagic shock despite successful resuscitation protocols^3^. The present clinical trial is the first study to investigate the immunomodulatory effects of remote ischemic conditioning in trauma patients sustaining hemorrhagic shock. Based on the salutary effects of RIC in experimental models of ischemia/reperfusion injury and hemorrhagic shock/resuscitation, we hypothesized that RIC exerts anti-inflammatory effects in hemorrhagic shock patients during the acute phase of injury. We demonstrated that application of RIC in hemorrhagic shock patients upon arrival in the Emergency Room at our institution was feasible, safe, tolerable, and not associated with adverse outcomes. The RIC protocol employed in this study, namely four cycles of inflation/deflation on the thigh, inhibited baseline and stimulated ROS production in neutrophils when applied to healthy controls. However, in our cohort of trauma patients, we did not observe significant changes in neutrophil activation, plasma pro-inflammatory markers, and clinical outcomes in patients treated with RIC in comparison to the Sham treatment. Still, we observed that RIC prevented a significant increase in Th2 cytokines in Sham-treated trauma patients—an effect that may potentially prevent Th2 induced immunosuppression.

Ischemia/reperfusion injury is a pathological process caused by tissue ischemia followed by restoration of perfusion, which together initiate a cascade of cellular events culminating in the tissue injury due to the generation of oxidative stress, initiation of the cell death pathways and generation of pro-inflammatory molecules^22^. In the context of trauma-induced hemorrhagic shock, post-trauma resuscitation induces ischemia/reperfusion in tissues and contributes to systemic inflammation and organ dysfunction. The extravasation and activation of neutrophils play a key role in the pathogenesis of organ injury through degranulation and the release of injurious enzymes and ROS. Consistent with prior reports, we observed priming of neutrophils, increased surface adhesion molecule expression, and degranulation of MPO during the early injury period concomitant with an early rise in the immunomodulatory cytokine IL-6 and chemokine IL-8 in our cohort of trauma patients. We asked the question whether RIC application in trauma-induced hemorrhagic shock patients might alter this pro-inflammatory profile, along with neutrophil ROS production and CD11b expression, both unstimulated and in response to PMA and fMLP. In the current study, the application of RIC following traumatic injury did not exert effects on these parameters and specifically, at no time point did neutrophil ROS production and CD11b expression differ, whether unstimulated or in response to PMA, between RIC and sham groups. We also observed that there was lower unstimulated and fMLP stimulated ROS production in both Sham and RIC treated patients compared to healthy controls, and a gradual decline in PMA stimulated ROS production, which normalized by 24 h post intervention for both groups. This may have been due to the elaboration of IL-10. This anti-inflammatory cytokine, known to exert inhibitory effects on neutrophil ROS production and phagocytic activity^23^, was elevated in both sham and RIC trauma patients throughout the study compared to healthy controls.

In addition to degranulation, activated neutrophils have also been shown to secrete VEGF^24^. Patients in the Sham group exhibited significant increases in plasma VEGF levels at 24 h post intervention, but this increase was not observed in the RIC group. The role of VEGF post injury remains controversial due to its dual role in inducing endothelial permeability and angiogenesis^25^. However, VEGF is associated with increased vascular permeability in patients with acute respiratory distress syndrome.

In our investigation of a panel of cytokines/chemokines, we observed that RIC prevented the significant increases in the cytokines MDC/CCL22 and TARC/CCL17 at 24 h post intervention. Both MDC and TARC are specific ligands for C–C chemokine receptor type 4, a receptor expressed on myeloid cells and lymphocytes but predominately on Th2 cells^26^. In addition to activating the Th2 response, TARC inhibits generation of classically activated macrophages resulting in impaired innate immunity^27^. Our findings suggest a potentially novel role for RIC in the modulation of T cell mediated innate immunity, as post traumatic shift from Th1 towards an anti-inflammatory Th2 phenotype contributes to susceptibility to nosocomial infections^6^ and development of sepsis and poorer outcomes. This suggestion is further supported by recent animal models, where RIC elicited increased T cell anti-inflammatory cytokine production with an attendant shift from Th17 cells towards Treg cells^28^. Importantly, Richter et al. revealed a role for MDC in mediating lung inflammation in a murine model of hemorrhagic shock/resuscitation as administration of neutralizing antibodies against MDC significantly reduces pulmonary levels of the chemotactic cytokines KC, MIP-2, and MIP-1α and extravasation of inflammatory cells^29^. Another potential beneficial effect of inhibiting MDC and TARC during the late resuscitation period is that MDC and TARC stimulate platelet aggregation^30^, which may induce thrombosis post trauma. The upregulation of antioxidants may play a role in the down regulation of MDC and TARC. Jeong and colleagues demonstrated that induction of HO-1 prevents IFN-γ and TNF-α-induced production of TARC and MDC through suppression of NF-κB in human keratinocytes^31^. Notably, induction of the antioxidant heme oxygenase-1 by RIC mediates lung protection in a rodent model of hemorrhagic shock/resuscitation^18^. We have also recently shown in a murine model that Nrf2, the upstream transcription factor for antioxidant response, mediated hepatoprotection by RIC after hemorrhagic shock/resuscitation^32^. In this trial, we did not observe any differences in the incidence of nosocomial infections or clinical outcomes between the Sham and RIC group, although our trial is not powered to detect such differences. Further studies are warranted to characterize T-cell phenotype and the innate immunity after RIC treatment post trauma.

Several studies in humans have sought to determine the potential mediators of RIC and have measured a number of humoral and immunomodulatory molecules, including plasma levels of cytokines, hormones, and factors^33–36^. These findings are summarized in Supplementary Table 2. Notably, Gedik et al. identified increased plasma IL-1α as a potential cardioprotective factor of RIC that protects the myocardium during ischemic cardioplegic arrest^34^. Although IL-1α was measured in our study, analysis was excluded as < 80% of samples had detectable levels. The study by Dewitte et al. found RIC increased plasma levels of antioxidant superoxide dismutase in a model of vascular injury in healthy volunteers^36^. Our present study did not measure superoxide dismutase, but this finding supports our prior animal study that determined the role of antioxidants in RIC-mediated protection^32^.

### Limitations

Despite a wealth of preclinical evidence pointing to the therapeutic effect of RIC in ischemia/reperfusion injury, translation to the clinical setting has been difficult^37^. Large prospective multicentre clinical trials have failed to demonstrate the efficacy of RIC in improving outcomes in the settings of cardiovascular surgery^38^ and acute myocardial infarction^39^. Several factors have been postulated that may negate the effects of RIC. These include surgical anesthesia, co-morbidities, age, as well as timing, route, and dose of RIC administration^10,40^. Many of these factors were pertinent in our trial and may have contributed to the neutral results of our study.

The nature of traumatic injuries and patient triage/transfer to our trauma centre affected our ability to precisely control the timing of initial RIC application across all patients as it relates to time after injury. Patients from our study had a median time of application at 2 h after injury, likely representing the post-resuscitative phase of injury. While there is no definitive clinical evidence to suggest the delayed application of RIC influences its protective effect, animal studies from our lab have shown that delaying RIC after shock/resuscitation in the early reperfusion period can diminish its protective effect^17^. Therefore, future trials on RIC might focus on pre-hospital application of RIC or try to standardize the intervention by applying RIC as soon as patients arrive to the trauma bay. One interesting patient population might be soldiers at risk of traumatic injuries who may have RIC applied prophylactically (preconditioning) during pre-deployment^41^. Any of these strategies might serve to maximize RIC’s protective effect.

The heterogeneity in trauma patients results in a number of clinical characteristics that significantly differ between each patient. These include the number of blood products transfused, surgical procedures, and anesthesia regimens. Each of these variables can contribute to a different immunological response^6,42^. Anesthesia is a major factor that may confound with the effects of RIC. For example, propofol has been shown to inhibit the cardioprotective effect of RIC in cardiac surgery^43^, whereas volatile anesthesia and opioids mimic the protective effects of RIC. The use of anesthesia was largely variable across our patient population and presents as a limitation in our study.

Previous studies in healthy volunteers that demonstrated the anti-inflammatory effect of RIC on leukocyte gene expression utilized the arm to conduct RIC^9^. Our current study utilized the thigh to induce RIC to permit the clinical resuscitation protocols performed on trauma patients. Controversy exists regarding the effectiveness of arm RIC versus leg RIC although a recent study by Dezfulian et al. demonstrated no significant differences in the liberation of nitrite—an effector circulating molecule of RIC, and the downstream cytoprotection induced by either method^44^. We showed in our healthy controls that thigh RIC induced down regulation of neutrophil oxidative burst and adhesion molecule expression, indicating the RIC stimulus applied on the leg reproduced the anti-inflammatory effect as forearm RIC. One potential explanation for reduced effect of RIC in trauma patients is that endothelial dysfunction, which develops in the early injury period^45^, has been shown to inhibit the generation of nitrite following reactive hyperemia^46^ and may therefore contribute to reduced generation of cytoprotective molecules by RIC. The significant reduction in peripheral blood flow to the limbs during hemorrhagic shock may also impair the initiation of signaling in the periphery by RIC. It is also unknown which dose (cycles) of RIC evokes optimal protective effects in humans as no dose response trial has been conducted^10^.

Activation of the vago-splenic axis has been shown by Lieder et al. to be causally involved in cardioprotection by RIC, implicating the spleen as a critical relay organ for its protective signaling^47^. In addition to severely impaired perfusion of the spleen during hemorrhage, splenic immune response and cellular immunity are further suppressed^48^, which together may potentially impair RIC’s protective signaling in trauma patients. To determine if arterial blood pressure during onset of RIC influences its effect, we performed post hoc analysis on subgroups of patients using surrogate markers of severe hemorrhagic shock (transfusion of blood products or no transfusion; injury severity score below or over 15; and admission systolic blood pressure below or over 90 mmHg) and found that these subgroups did not impact RIC on the biomarkers measured in this study. Traumatic injuries also impair platelet function, as platelets become inert to ex vivo stimulation, in a condition known as platelet exhaustion^49^. Lieder et al. recently demonstrated that platelets play a causal role in RIC signaling^50^. Thus, platelet dysfunction as a consequence of trauma may contribute to undermine RIC’s signaling.

In summary, our study was the first trial to investigate the immunomodulatory effect of RIC in humans sustaining traumatic hemorrhagic shock. We demonstrate that post-trauma application of RIC in hemorrhagic shock patients is safe, feasible and not associated with adverse outcomes. Although RIC induced anti-inflammatory effects in neutrophils taken from healthy volunteers, this effect was not recapitulated in trauma patients, which may in part be due to confounding factors, including the timing of the intervention. However, RIC prevented the increase in Th2 cytokines that are associated with immunosuppression. Results from our preliminary study in trauma patients in hemorrhagic shock suggest further investigation into the possible immunomodulatory mechanisms of RIC on the post-trauma immuno-inflammatory response and its impact on clinical parameters.

## Supplementary Information

Supplementary Information 1.

Supplementary Information 2.

## Data Availability

All data generated or analyzed during this study are included in this published article [and its supplementary information files].
