# Supplementary material for: Effect of remote ischemic conditioning on the immune-inflammatory profile in patients with traumatic hemorrhagic shock in a randomized controlled trial
Source: Sci Rep. 2023 Apr 29;13:7025. doi: 10.1038/s41598-023-33681-3 (PMC10148877; doi:10.1038/s41598-023-33681-3)
Supplement: Supplementary file 2 — Supplementary Information 2. [file 41598_2023_33681_MOESM2_ESM.pdf]

Supplementary Table 1. Plasma MPO, cytokine, and chemokine levels in Healthy Controls.

	Admission	1 hour	3 hour	24 hour
**MPO (pg/mL)**				
**Healthy-Sham**	29.96 (22.98–34.78)	31.17 (–23.64–32.42)	33.59 (25.06–36.52)	27.71 (23.89–34.87)
**Healthy-RIC**	28.79 (24.14–47.45)	31.86 (25.86–44.28)	33.40 (28.96–45.67)	32.42 (26.55–47.69)
**IL-6 (pg/mL)**				
**Healthy-Sham**	0.28 (0.00–0.59)	0.41 (0.28–0.74)	0.36 (0.16–0.38)	0.51 (0.16–0.63)
**Healthy-RIC**	0.00 (0.00–0.00)	0.43 (0.00–0.60)	0.47 (0.13–0.54)	0.00 (0.00–0.21)
**TNF-α (pg/mL)**				
**Healthy-Sham**	1.76 (1.72–1.97)	1.74 (1.59–2.13)	1.82 (1.51–2.10)	1.84 (1.50–1.85)
**Healthy-RIC**	2.01 (1.73–2.24)	1.93 (1.51–2.11)	1.76 (1.43–2.06)	2.08 (1.58–2.18)
**IL-10 (pg/mL)**				
**Healthy-Sham**	0.31 (0.26–0.31)	0.30 (0.26–0.43)	0.34 (0.26–0.46)	0.29 (0.25–0.42)
**Healthy-RIC**	0.27 (0.20–0.36)	0.28 (0.23–0.33)	0.30 (0.26–0.33)	0.25 (0.21–0.34)
**IL-15 (pg/mL)**				
**Healthy-Sham**	2.26 (1.89–2.68)	2.45 (2.24–4.16)	2.46 (2.33–3.79)	2.25 (2.01–3.08)
**Healthy-RIC**	1.99 (1.76–3.43)	3.16 (2.84–5.45)	3.53 (1.29–4.98)	2.04 (1.51–3.34)
**IL-16 (pg/mL)**				
**Healthy-Sham**	134.76 (127.45–152.24)	189.63 (149.41–194.89)	193.64 (125.21–213.71)	167.23 (114.55–212.41)
**Healthy-RIC**	172.95 (137.92–198.69)	223.35 (154.26–684.09)	215.39 (79.57–1473.43)	181.71 (169.56–193.16)
**IL-17A (pg/mL)**				
**Healthy-Sham**	2.50 (1.79–3.84)	1.54 (0.00–2.22)	1.79 (0.85–4.19)	3.02 (0.96–3.84)
**Healthy-RIC**	1.57 (1.18–2.10)	2.04 (0.91–4.48)	0.00 (0.00–2.08)	1.19 (0.00–1.99)
**IL-12 p40 (pg/mL)**				
**Healthy-Sham**	124.70 (76.45–150.05)	101.37 (66.22–114.14)	105.61 (56.78–165.18)	105.92 (75.33–188.50)
**Healthy-RIC**	100.29 (78.77–130.64)	99.55 (62.12–131.26)	71.64 (34.59–140.20)	108.79 (75.01–122.86)
**IFN-γ (pg/mL)**				
**Healthy-Sham**	5.43 (3.67–6.27)	5.50 (3.67–5.58)	4.36 (4.28–5.69)	3.96 (3.80–5.56)
**Healthy-RIC**	4.84 (4.13–8.23)	4.97 (3.99–7.96)	4.81 (3.95–8.93)	5.18 (3.21–6.31)
**IL-5 (pg/mL)**				
**Healthy-Sham**	0.90 (0.64–0.91)	1.44 (0.78–2.03)	0.87 (0.76–1.56)	0.67 (0.62–0.71)
**Healthy-RIC**	0.58 (0.28–1.07)	0.96 (0.60–1.31)	1.06 (0.70–1.29)	0.66 (0.46–0.93)

**IL-7 (pg/mL)**				
**Healthy-Sham**	2.52 (1.26–2.98)	2.39 (1.42–4.78)	1.73 (0.86–2.11)	3.21 (2.01–3.51)
**Healthy-RIC**	2.21 (1.72–2.97)	2.14 (1.63–2.87)	1.63 (0.56–3.06)	2.71 (1.13–3.96)
**VEGF (pg/mL)**				
**Healthy-Sham**	20.27 (15.28–24.96)	19.29 (7.23–22.93)	20.42 (11.94–25.49)	19.97 (12.51–24.41)
**Healthy-RIC**	15.71 (14.26–24.59)	14.28 (10.97–26.18)	6.89 (3.31–20.58)	17.92 (11.79–21.65)
**MDC (pg/mL)**				
**Healthy-Sham**	655.05 (572.49–919.92)	683.88 (560.83–756.85)	630.11 (560.87–674.14)	577.33 (513.49–830.08)
**Healthy-RIC**	669.09 (568.23–932.30)	880.78 (567.36–961.33)	869.40 (598.95–914.89(	926 (631.19–979.74)
**TARC (pg/mL)**				
**Healthy-Sham**	41.69 (33.84–43.10)	37.09 (24.99–40.39)	37.14 (23.70–38.23)	44.85 (30.58–52.83)
**Healthy-RIC**	31.83 (25.08–58.11)	21.99 (17.11–43.86)	23.13 (21.60–35.41)	27.98 (20.58–52.05)
**MIP-1β (pg/mL)**				
**Healthy-Sham**	48.50 (29.70–49.97)	42.95 (26.85–53.07)	44.27 (26.46–49.71)	51.27 (25.91–65.88)
**Healthy-RIC**	30.30 (28.15–34.15)	30.65 (26.50–36.84)	30.54 (28.49–36.30)	28.23 (28.07–38.70)
**MCP-1 (pg/mL)**				
**Healthy-Sham**	58.85 (57.57–72.36)	52.78 (46.86–70.70)	56.14 (51.62–69.30)	59.15 (49.53–64.94)
**Healthy-RIC**	53.27 (45.40–62.07)	52.35 (44.22–69.37)	55.50 (47.61–76.00)	58.68 (49.09–69.53)
**IL-8 (pg/mL)**				
**Healthy-Sham**	2.86 (2.29–3.42)	2.22 (1.83–2.64)	2.32 (1.92–2.57)	3.19 (2.20–3.21)
**Healthy-RIC**	1.72 (1.15–2.44)	1.43 (0.81–1.96)	1.84 (1.79–2.06)	2.09 (1.52–3.10)
**Eotaxin (pg/mL)**				
**Healthy-Sham**	96.82 (77.69–115.77)	83.50 (63.95–131.85)	79.68 (68.78–146.04)	81.41 (71.34–123.14)
**Healthy-RIC**	58.71 (46.82–110.16)	65.55 (57.61–120.57)	88.72 (64.02–96.80)	60.27 (48.09–136.46)
**IP-10 (pg/mL)**				
**Healthy-Sham**	292.51 (181.87–363.47)	344.70 (199.22–353.28)	328.83 (197.56–386.42)	255.96 (176.72–270.80)
**Healthy-RIC**	206.39 (182.56–294.04)	236.31 (190.61–324.22)	235.33 (190.15–323.29)	255.01 (190.37–254.71)
**MCP-4 (pg/mL)**				
**Healthy-Sham**	31.71 (20.83–35.47)	27.68 (22.04–29.35)	23.29 (18.98–25.86)	36.52 (20.56–41.48)
**Healthy-RIC**	27.28 (25.46–46.57)	20.14 (18.19–42.73)	23.68 (17.59–47.28)	39.58 (21.41–54.08)

Supplementary Table 1. Plasma levels of myeloperoxidase (MPO), cytokines, and chemokines in healthy controls. Values represent median (interquartile range).

Supplementary Table 2. Clinical studies on RIC evaluating plasma levels of humoral mediators.

Study	Study Setting	Outcome Measures (plasma levels)	Findings
Nielsen et al[33]	Kidney transplant	CXCL11, GM-CSF, CX3CL1, INFγ, IL-10, MIP3α, IL-12, IL-13, IL-17a, IL-1β, IL-2, IL-21, IL-4, IL-23, IL-5, IL-6, IL-7, IL-8, MIP1α, MIP1β, TNF-α	No significant changes
Hummitzsch et al[35]	Healthy volunteers	CXCL5, Growth hormone, IGFBP3, IL-1α, IL-6, Angiopoietin 2, VEGF, PECAM-1, sTie-2, IL-8, MCSF	No significant changes
Dewitte et al[36]	Vascular injury in healthy volunteers	SDF-1α, MCP-1, ADMA, VEGF, SOD	SOD significantly increased
Gedik et al[34]	Coronary artery bypass graft surgery	Apo-A1, EPO, GDF-11, GHRH, GHRP, GLP-1, GH, HIF-1α, IL-1α, IL-1β, IL-2, IL-6, IL-8, IL-10, IL-15, IL-17, IL-33, leptin, pentraxin-3, prolactin, RNase-1, SDF-1α, survivin, thymosin-β4, TNF-α	IL-1α significantly increased

Supplementary Figure 1A

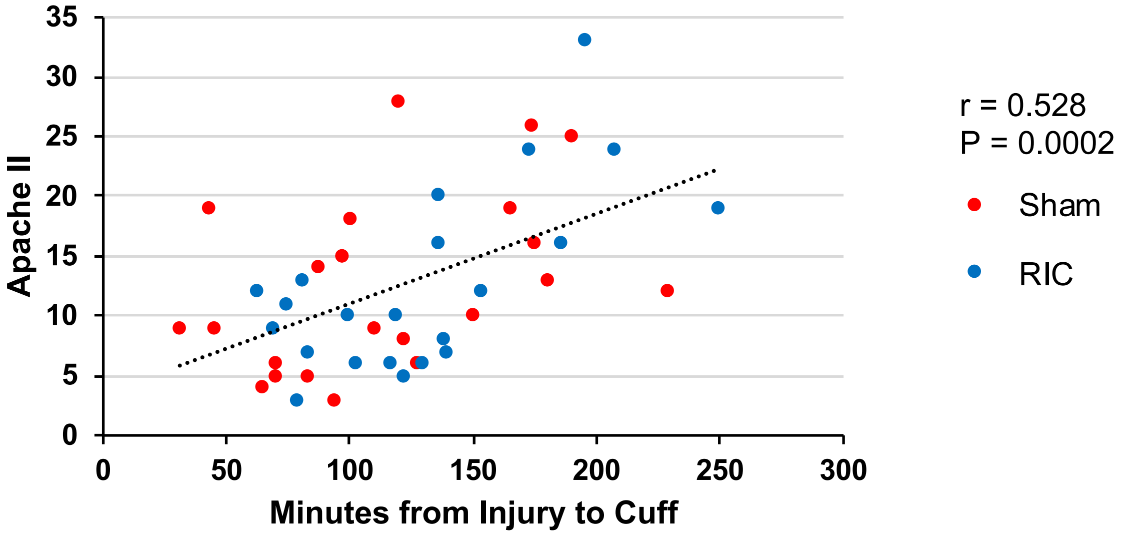

Supplementary Figure 1B

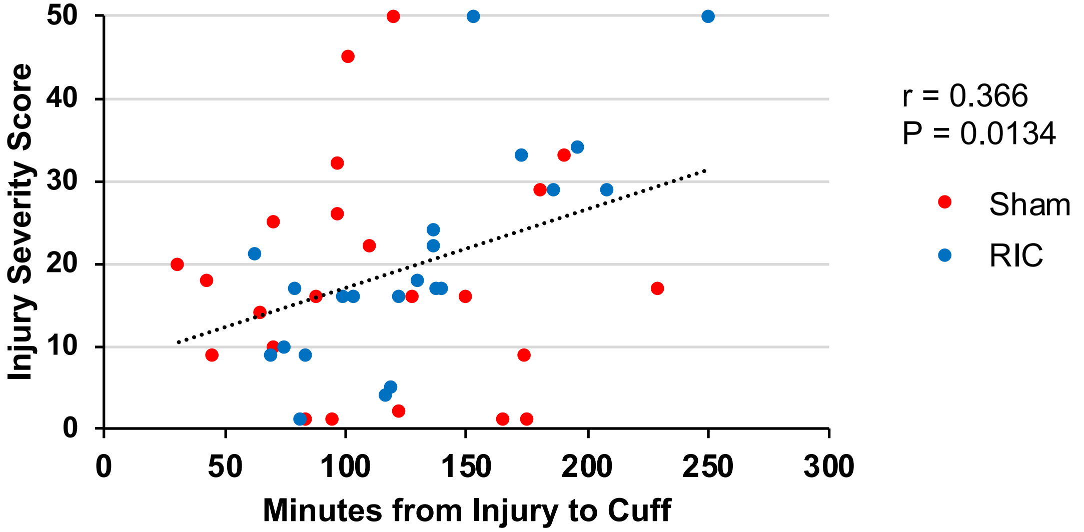

Supplementary Figure 1: Pearson's correlation comparing APACHE II score to the duration from the time of injury to application of Sham or RIC interventions. B. Pearson's correlation comparing Injury Severity Score to the duration from the time of injury to application of Sham or RIC interventions.

Red circle = Sham; Blue circle = RIC

Trauma-Sham
Trauma-RIC

* P < 0.05 vs Healthy
** P < 0.01 vs Healthy

a P < 0.05 vs Sham Admission
b P < 0.01 vs Sham Admission

c P < 0.05 vs RIC Admission
d P < 0.01 vs RIC Admission

Suppl Fig 2A

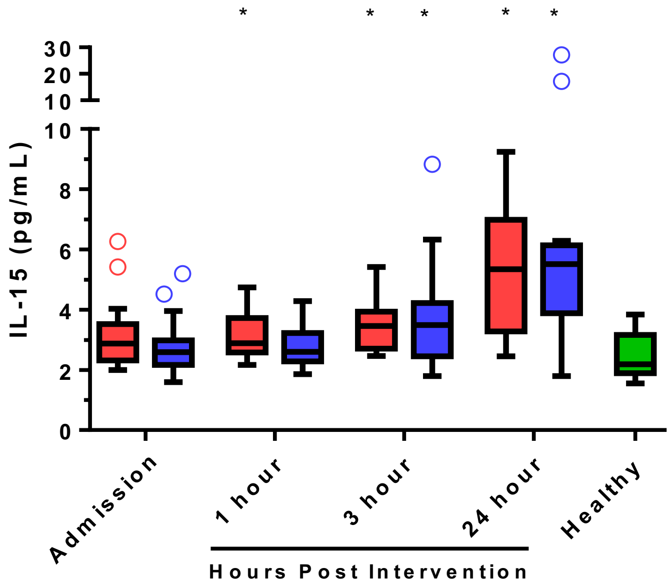

Suppl Fig 2B

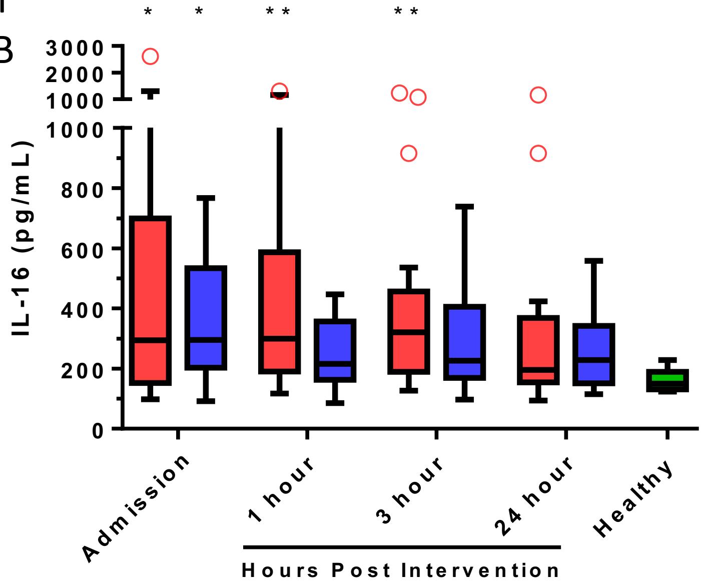

Suppl Fig 2C

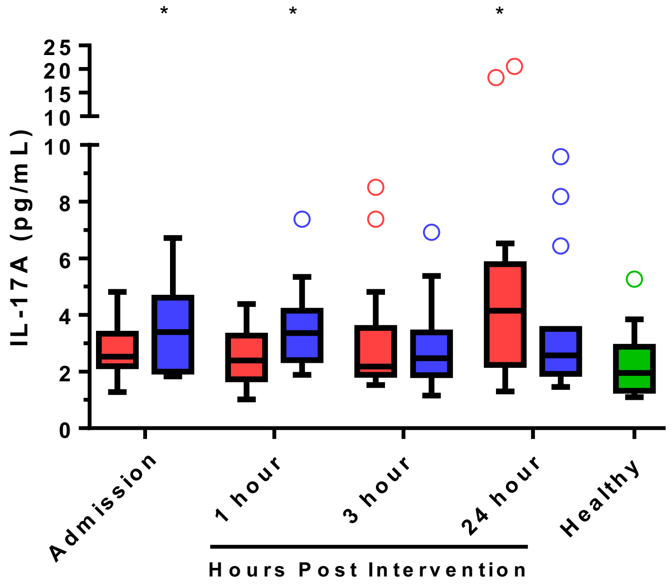

Suppl Fig 2D

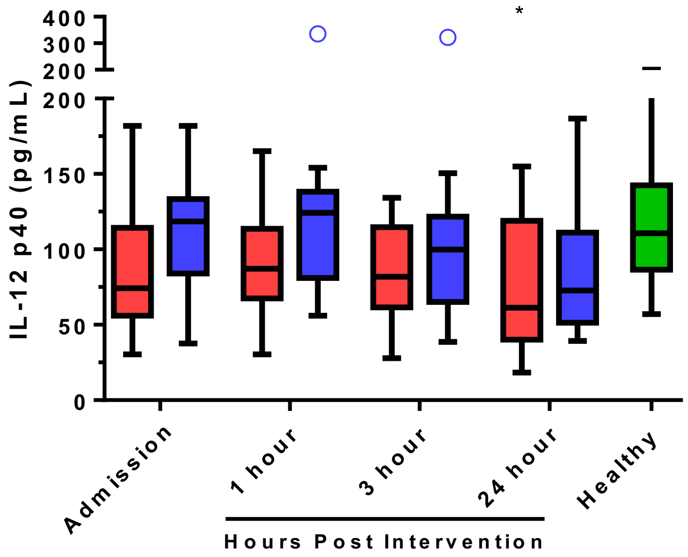

Suppl
Fig 2E

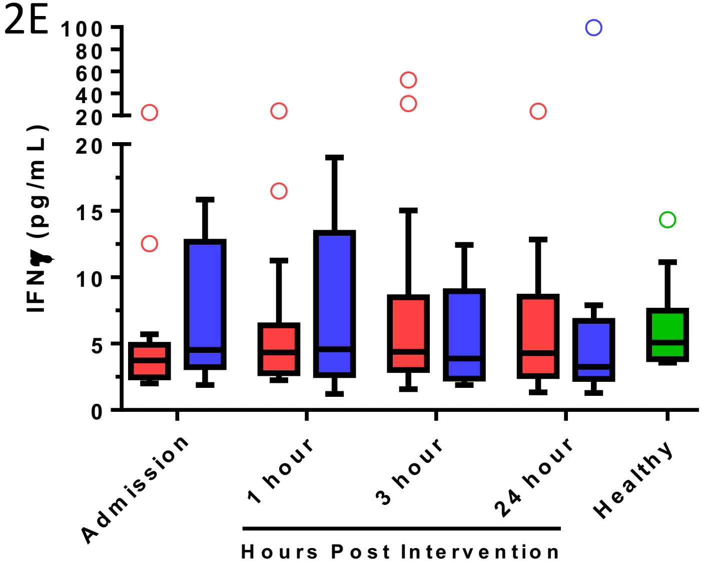

Suppl
Fig 2F

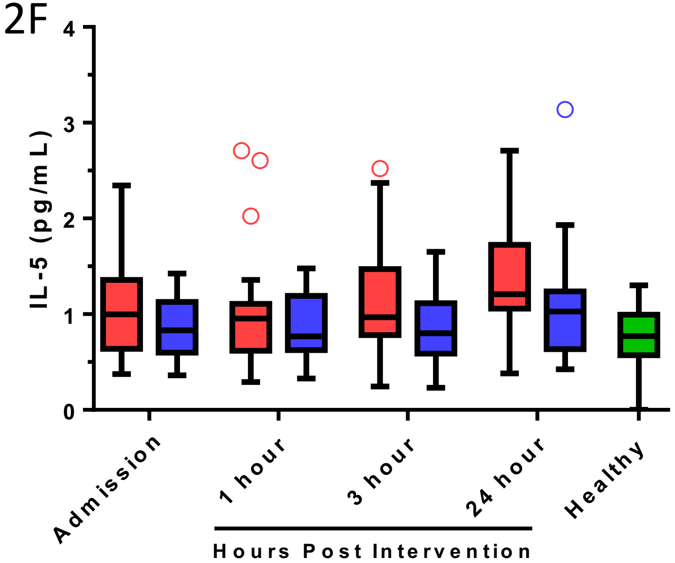

Suppl
Fig 2G

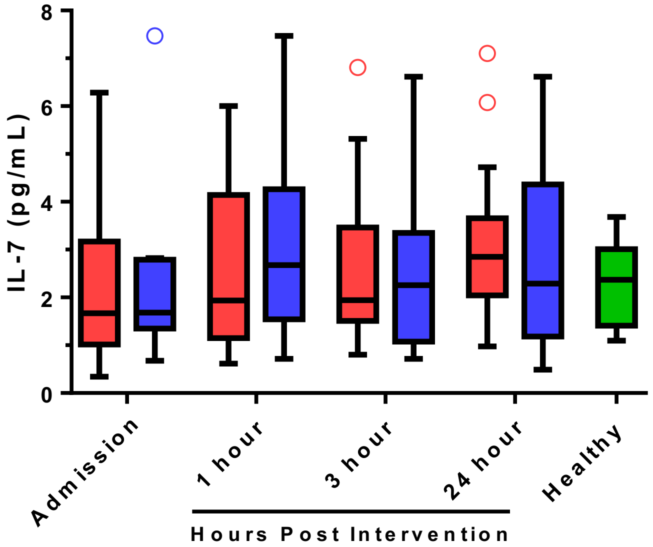

Suppl
Fig 2H

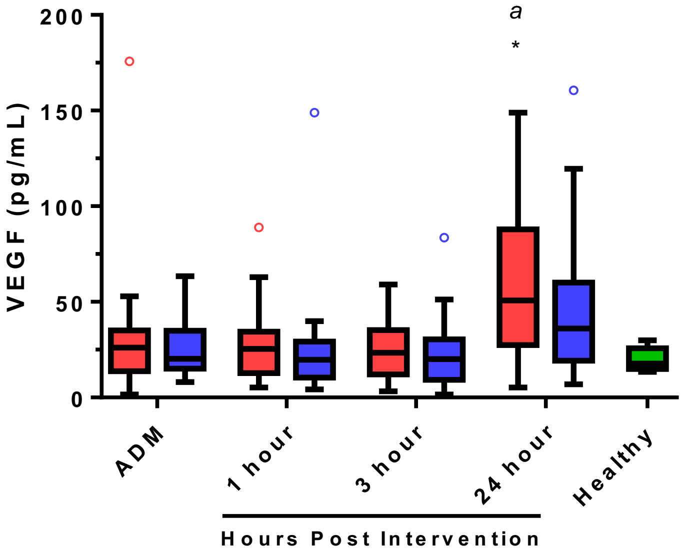

Supplementary Figure 2: Plasma cytokine levels in trauma patients. A. IL-15 B. IL-16 C. IL-17 D. IL-12p40 E. IFN-γ F. IL-5 G. IL-7 H. VEGF.

Red open circle = Trauma-Sham. Blue open circle = Trauma-RIC. Green open circle = Healthy Controls. Lines represent the median and interquartile range. *a* $P < 0.05$ vs Sham Admission. *b* $P < 0.01$ vs Sham Admission. *c* $P < 0.05$ vs RIC Admission. *d* $P < 0.01$ vs RIC Admission. * $P < 0.05$ vs Healthy Controls. ** $P < 0.01$ vs Healthy Controls.

Suppl
Fig 3A

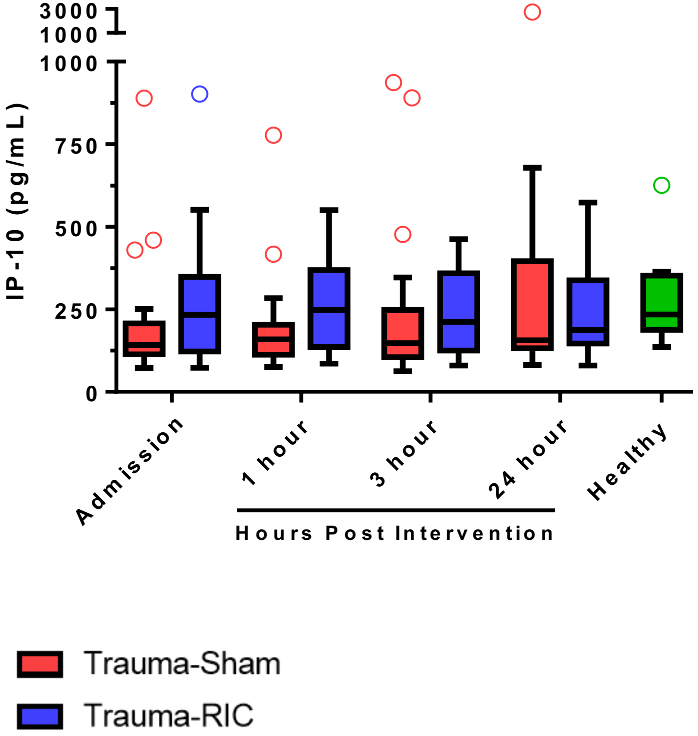

Suppl
Fig 3B

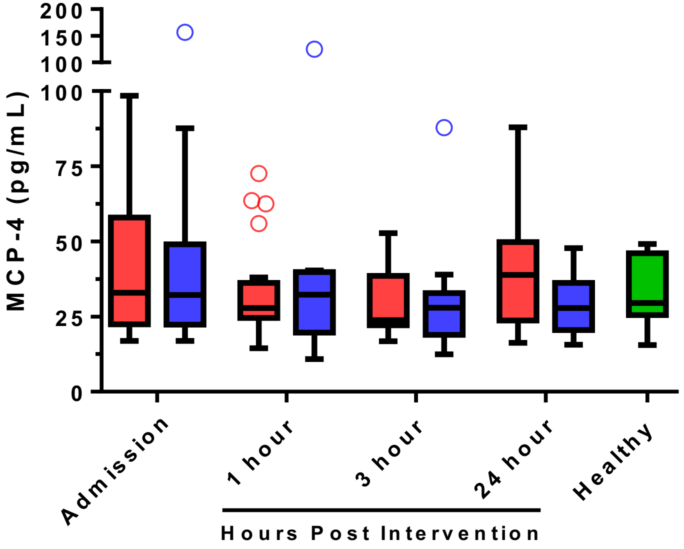

Supplementary Figure 3. Plasma chemokine levels in trauma patients. A. IP-10 B. MCP-4

Red open circle = Trauma-Sham. Blue open circle = Trauma-RIC. Green open circle = Healthy Controls. Lines represent the median and interquartile range.
